# Book Notices

**Published:** 1882-04-20

**Authors:** 


### *BOOK NOTICES.*

A TREATISE ON DISEASES OF THE EYE. By Henry D. Noyes, A. M., M. D., Professor of Ophthalmology and Otology, in Bellevue Hospital Medical College; Surgeon to the New York Eye and Ear Infirmary; President of the American Ophthalmological Society; Member of the New York Ophthalmological Society; Permanent Member of the Medical Society of the State of New York; Member of the New York Academy of Medicine. New York: William Wood & Co. McGarity & Laird, Agents, Atlanta, Ga. Illustrated—354 large octavo pages.

The general anatomy and physiology of the globe very plain and satisfactory. The various diseases and operations admirably handled and brought down to recent and well approved principles.

LECTURES ON DISEASES OF CHILDREN, A HANDBOOK FOR PHYSICIANS AND STUDENTS. By Dr. Edward Henoch, Director of the Clinic and Polyclinic for Diseases of Children, in the Royal Charete, and Professor in the University of Berlin. New York: William Wood & Co., 1882.

This work contains 357 octavo pages, and though styled a Handbook, is sufficiently full on many subjects treated. This, however, cannot be said of Cholera Infantum and Diarrhea, the most frequent and fatal of infantile affections, the remarks upon which are somewhat meagre, and contain no new or important suggestions. Its brevity, however, and its avoidance of detail upon subjects already fully considered in the many treatises upon this department, will commend the work to many readers. The lucture style will prove attractive, as also the numerous cases from the author's experience.

A SYSTEM OF SURGERY, THEORETICAL AND PRACTICAL, IN TREATISES, BY VARIOUS AUTHORS. Edited by T. Holmes, M. A. Cantab., Surgeon and Lecturer on Surgery at St. George's Hospital; Memb. Corresp. De La Societe De Chirurgie De Paris. First American from Second English edition, thoroughly revised and much enlarged, by John H. Packard, A. M., M. D., Surgeon to the Episcopal and St. Joseph's Hospitals, Philadelphia. Assisted by a large corps of the most eminent American Surgeons, in three volumes. Vol. II. Diseases of Organs of Special Sense; Diseases of Circulatory System; Diseases of the Genito-Urinary Organs. Philadelphia: Henry C. Lea's Sons & Co., 1881.

The above is the second volume of the great Surgical work, and contains 1063 double column pages. A list is given of the numerous eminent contributors, both American and English. The illustrations are numerous, and some of them beautifully colored. As the work is ample in its treatment of the Organs of Special Sense, it is suited to the library of the Specialist as well as of the General Surgeon. It is full, plain and able upon all the subjects treated, and must be regarded as a very valuable work to the Surgeon for its practical features, and to the Physician as a work of reference upon many subjects upon which information may be sought.

Volume III. of the same work is also just received—same style and size—numbering 1059 pages, elegantly gotten up—illustrated, and treats of Diseases of Respiratory Organs, Diphtheria, Croup, Diseases of Larynx, Diseases of the Thyroid body—Apnœa.

A TREATISE ON HUMAN PHYSIOLOGY, DESIGNED FOR THE USE OF THE STUDENT AND PRACTITIONERS OF MEDICINE. By John C. Dalton, M. D., Professor of Physiology and Hygiene in the College of Physicians and Surgeons, New York; Member of the New York Academy of Medicine; of the New York Pathological Society; of the American Academy of the Arts and Sciences, Boston; of the Biological Department of the Academy of Natural Sciences, Philadelphia, and of the National Academy of Sciences of the United States of America Seventh edition, with two hundred and fifty-two illustrations. Philadelphia. Henry C. Lea's Sons & Co, 1882.

This new and revised edition of this excellent and popular text-book of Physiology will be welcomed by the profession, and especially by Medical Students. We note important and useful improvements in the present work, which will add to its popularity as a text-book; improvements which bring it well up with recent advances. The alterations relating to the classification of Albuminoids, and the additions in regard to Ferments are interesting and presented in a manner at once plain and intelligible.

The department of Physiological Chemistry is also well and fully considered, and facts drawn from the interesting observations, which of late years have been attracting attention in respect to the localization of the cerebral functions, are in the present work more fully and satisfactorily discussed than in previous editions. We have no hesitation in pronouncing this edition as an improvement, and the work as now presented, as among the best which has yet been published on this interesting branch of Medical Science.

ILLUSTRATIONS OF DISSECTIONS IN A SERIES OF ORIGINAL COLORED PLATES THE SIZE OF LIFE, REPRESENTING THE DISSECTION OF THE HUMAN BODY. By George Virner Ellis, Professor of Anatomy in University College, London, and J. H. Ford, Esqr. The drawings are from nature, by Mr. Ford, from dissections by Professor Ellis. Reduced on a uniform scale, and reproduced in fac simile, expressly for Wood's Library of Standard Medical Authors. Vol. I. Second edition. Pages 253, octavo. New York: William Wood & Co. McGarlty & Laird, Agents, Atlanta, Ga.

To the Student of Anatomy, this is a very valuable work. The illustrations are from real life, and present only such dissections as are commonly seen in the practical Anatomy room. In the present volnme are presented views from the head and neck, the upper extremities, the abdominal parietes and the lower limbs.

LECTURES ON ELECTRICITY, [DYNAMIC AND FRANKLINIC] IN ITS RELATIONS TO MEDICINE AND SURGERY. By A. D. Rockwell, A. M., M. D., Electro-Therapeutist to the New York State Woman's Hospital; Member of the American Neurological Association; Fellow of the New York Academy of Medicine; Member of the New York County Medical Society, etc. 122 pages, large octavo.

This work is interesting and instructive, and presents a number of new subjects, among which are descriptions of the Galvanic Accumulator for storing electricity for use, and the induction balance for locating bullets in the body. The several methods of franklinization and its value as compared to dynamic electricity.

HOMEOPATHY—WHAT IS IT? A STATEMENT AND REVIEW OF ITS DOCTRINES AND PRACTICE. By A. D. Palmer, M. D., LL. D., Professor of Pathology and Practice of Medicine in the College of Medicine and Surgery in the University of Michigan. Second edition, 1881. Detroit, Michigan: Geo. S. Davis, Publisher.

This little work by Dr. Palmer, has attracted much attention by the profession, and called down many anathamas by the Homeopathists. It should be read by every practitioner.

